# The use of a woman’s own eggs in her first IVF treatment at the age of 48 years and 10 months with successful live birth after PGT-A: a case report

**DOI:** 10.5935/1518-0557.20240072

**Published:** 2025

**Authors:** Claudia Gomes, Mariana Nicolielo Barreto, Anaíde Silva Sousa, Dayane Guerino Reis, Priscila Beirigo, Izadora Reis, Caroline Fauth, Andrea Belo, Hanna Park, Ana Luiza Tavares, Livia Munhoz, José Roberto Alegretti, Dóris S. Chéles, Aline R. Lorenzon

**Affiliations:** 1 Clinical Department, Huntington Centro de Medicina Reprodutiva - Eugin Group, São Paulo, SP, Brazil; 2 Embryology Laboratory, Huntington Centro de Medicina Reprodutiva - Eugin Group, São Paulo, SP, Brazil; 3 Research & Development Department, Huntington Centro de Medicina Reprodutiva - Eugin Group, São Paulo, SP, Brazil

**Keywords:** oldest woman, IVF, own eggs, euploid embryo, case report

## Abstract

Natural conception in women after the age of 45 years is rare. The probability of successful pregnancy in this specific group of women after IVF and embryo transfer with autologous oocytes is also reduced. In addition, advanced maternal age is associated with an increased risk of aneuploidies and other associated complications during pregnancy. Generally, women who are over 44 years old are advised to receive IVF treatment with donated oocytes due to poor oocyte quality and low ovarian reserve. Although IVF outcomes in women of advanced age can be associated with the best prognosis when donated oocytes are used, IVF is not always well accepted by infertile couples. This is a case report of a woman who achieved a clinical pregnancy and live birth in her first attempt at IVF treatment with her own eggs and a euploid embryo at 48 years and 10 months, respectively, at the time of oocyte retrieval. This case demonstrates that limited attempts at assisted reproductive technology with older women’s own eggs may be an option in specific cases.

## CASE REPORT

In November 2022, a 48-year-old woman and 51-yearold man were admitted to our clinic due to their advanced age to investigate the possibility of having a third child. They had had two children who were 17 and 14 years old who were conceived spontaneously (gravidity 2, parity 2) and delivered by C-section on maternal request, without gestational complications in either pregnancy. The couple had not tried to conceive again after the second child was born. The death of the oldest child in October 2022 in a motorcycle accident led the couple to the decision to seek reproductive medical assistance. The woman had a medical history of idiopathic hyperprolactinemia since 19 years of age and hypothyroidism, both of which were under control with medications, and no other disease was reported. She presented with a regular menstrual cycle of 25 days, with no dysmenorrhea, and apart from two elective C-sections, she underwent no other surgeries. Her anti-Müllerian hormone (AMH) level was 0.02 ng/mL, her antral follicular count was 5, and her thyroid-stimulating hormone and prolactin levels were normal. She had low free testosterone levels (0.98pg/mL). Her transvaginal ultrasound, Pap smear and mammography results were normal. Her body mass index was 21.15kg/m^2^.

Her partner’s medical history revealed treatment for previous nephrolithiasis, and it was the only surgery he had received. His body mass index was 26.51 kg/m^2^. The couple’s karyotypes were normal, and both were under psychological therapy and antidepressant medication due to their recent loss (clonazepan, trazadon and desvenlafaxin).

Even after medical counseling that scientific evidence on IVF treatment with autologous oocytes that results in pregnancy in 48-year-old women is extremely rare, the couple persisted with the desire to proceed with treatment. Antioxidant supplements to minimize age-related oxidative stress were prescribed for both, including 600 mg/day of coenzyme Q10 and 12.5 mg/day of testosterone gel for the woman, for 60 days before egg retrieval to increase follicular FSH responsiveness. On February 2023, when she was 48 years and 10 months old, five antral follicles and FSH-r 150 UI plus hMGl 150UI and dydrogesterone 10 mg twice a day were prescribed for ovarian stimulation and inhibition of the LH surge, respectively. On day nine of ovarian stimulation, oocyte maturation was triggered with hCG + a GhRh agonist (dual trigger). The male semen parameters before preparation were a total sperm count of 33x10^6^million/mL, a total sperm progressive count of 10x10^6^ million/ mL and a Kruger morphology of 2%. After sperm preparation with the Zymot Multi Fertility® device, the total number of sperm and the progressive count were 7x10^6^million/ mL, and Kruger’s morphology was 3%. Five eggs were retrieved-four at metaphase II (MII)-that were fertilized by ICSI, resulting in three blastocysts (4BB, 4AA, and 4BB, morphological grading according to Gardner and Schoolcraft (Gardner *et al.*, 2000) at day five of embryo culture in a time lapse incubator (Embryoscope Plus®, Vitrolife) and continuous single culture media (CSC-NX Complete®, Irvine Scientific).

**Figure 1:**
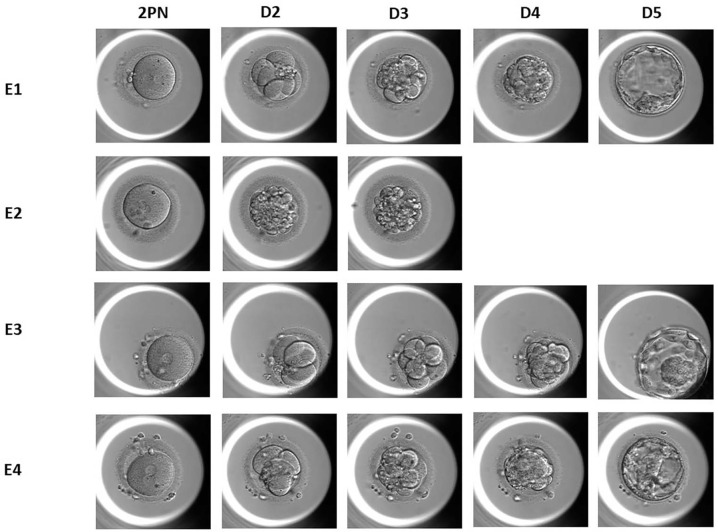
Embryo development (E1-E4) from fertilization (2PN) until blastocyst formation (Day 5) in a time-lapse incubator after ICSI in four freshly retrieved MII oocytes.

All three embryos were subjected to trophectoderm biopsy followed by preimplantation genetic testing for aneuploidy (PGT-A). Embryo #1 was reported as euploid (46XX), morphological grading according to Gardner and Schoolcraft 4BB (Gardner et al., 2000) and Vitrolife’s KIDScore 5.8 (Kato *et al*., 2018). The other two blastocysts were aneuploid (embryo #3, 47XX+7, 4AA, 8.1) and (embryo #4, 46XX -7-16-22 +9+13+20, 4BB, 5.6, respectively) (Figure 1).

On March 2023, uterine preparation was started on day two of the menstrual cycle with 2 mg of valerate estradiol twice a day for 3 days and then 4 mg of valerate estradiol twice a day for more than 3 days, but the endometrial lining was not adequate (hyperechogenic aspect), and progesterone levels increased prematurely; therefore, we cancelled embryo transfer.

On May 2023, an artificial cycle for frozen embryo transfer was conducted as followed: 2 mg of valerate estradiol every 8 hours was prescribed on day two of the menstrual cycle for 6 days, and 3mg of estradiol gel was added twice a day. After nine days of hormonal therapy, the endometrial lining was 8.1mm long, trilaminar and a dominant follicle was detected by ultrasound measuring 17 mm, estradiol levels were 539pg/mL, and progesterone levels were 0.2 ng/mL. No medication has been used to trigger oocyte release. Vaginal progesterone (800 mg/day) was prescribed, and embryo transfer was performed five days later when the patient was 49 years and 2 months old. Her estradiol level was 239pg/mL, and her progesterone level was 10.4ng/mL one day before embryo transfer. There were no technical difficulties with the embryo transfer procedure.

The first quantitative beta-hCG was measured ten days after embryo transfer, and the result was 216mUI/ mL. Luteal support was continued until the eighth week of gestation, when the medication dosage progressively decreased and completely stopped at twelve weeks of gestation. Firstand second-trimester morphological ultrasound data, including uterine artery Doppler data, were normal for all risk markers. The patient had an ongoing pregnancy without any complications, and acetylsalicylic acid (100mg/ day) was used for preeclampsia prevention. A healthy baby girl, with a birth weight of 2.902g and length of 49cm, was born in late February 2024 at 39.0 weeks of pregnancy by elective nonlabored C-section. The APGAR scores at 1 and 5 min were 9 and 10, respectively. The patient and baby were discharged from the hospital after 48 h.

Informed consent was obtained from the subjects of this case report.

## DISCUSSION

It is well known that female fertility declines with age. Fertility starts declining significantly at the age of 32 and declines progressively after 35 years, decisively worsening in the late 40s, prior to menopause (Ubaldi *et al.*, 2019; Mikwar *et al.*, 2020). Advanced maternal age, which is consensually attributed to women older than 35 years, is a physiological condition in which women have diminished rates of spontaneous conception and live birth following IVF, compromised ovarian follicular reserve and increased rates of embryo aneuploidies due to ovarian aging (Buratini *et al.*, 2022).

Social changes related to the new roles of women in the workplace in recent decades, together with the increase in life expectancy and the consolidation of conjugal affective relationships later in life, have contributed to the postponement of parenthood, especially in developed and developing countries (Montagnini *et al.*, 2023). These new aspects of society, together with a more prominent natural decline in fertility in women after 35 years of age, have led to an increase in the use of assisted reproductive techniques (SART - Society for Assisted Reproductive Technology, 2023) and especially an increase in IVF cycles involving egg donation. According to the Latin American Register Report, the proportion of cycles using egg donation was 10.2% in 2001 and 15.3% in 2020 (Zegers-Hochschild *et al.*, 2023).

Natural conception in women after the age of 45 years is rare. The probability of successful pregnancy in this specific group of women after IVF and embryo transfer with autologous oocytes is also reduced. In addition, advanced maternal age is associated with an increased risk of aneuploidies and other complications associated with pregnancy. Generally, women who are over 44 years old are advised to receive IVF treatment with donated oocytes due to poor oocyte quality and low ovarian reserve. Although IVF outcomes in women of advanced age can be associated with the best prognosis when donated oocytes are used, they are often not acceptable to all infertile couples (Rani *et al.*, 2015; Gleicher *et al.*, 2016).

Nevertheless, there are reported cases in which women successfully conceived through IVF with their own eggs in their later 40s (Rani *et al.*, 2015; Gleicher *et al.*, 2018; Wu *et al.*, 2022). Although rare, there are women who present favorable genetic and hormone profiles and who benefit from autologous IVF cycles.

This is a rare case report of a woman who became pregnant and achieved a live birth after using her own oocytes and having a euploid embryo at the age of 48 years and 10 months. In 2015, a 50-year-old Indian woman, considered the oldest IVF patient resulting in a live birth without preimplantation genetic testing for aneuploidies (PGT-A), was reported at the time of her oocyte retrieval. Her AMH was 1.74 ng/mL, and she had a history of hypertension and diabetes in addition to severe preeclampsia superimposed on chronic hypertension during pregnancy (Rani *et al.*, 2015).

A 47-year-old woman at the time of her oocyte retrieval was one of the oldest patients reported to have given birth recently without PGT-A screening (Wu *et al.*, 2022). Our patient had an AMH concentration of 0.02 ng/mL, which was even lower than that of a 47-year-old patient (0.24 ng/mL), demonstrating the challenge of achieving a successful pregnancy with limited ovarian reserve (Jirge, 2016; Busnelli *et al.*, 2022). Both of them had normal spontaneous deliveries before treatment, which may emphasize the relevance of a previous fertility history in these patients. According to Wu *et al.* (2022), one oocyte was retrieved, which resulted in a cleaved 8I embryo (Gardner’s embryo score system) (Gardner *et al.*, 2000). This embryo was frozen and then thawed one day before the uterine transfer. On the day of transfer, the embryo was classified as 10III (Gardner *et al.*, 2000). Patient pregnancy was confirmed 14 days after frozen-thawed embryo transfer. The male baby was born at 36 weeks and 5 days. Our patient transferred a euploid blastocyst, the oldest reported case to achieve a clinical pregnancy and live birth with her own eggs after PGT-A.

There is a consensus that in patients older than 42 years, there is a low success rate related to pregnancy and live birth following IVF. The pregnancy and live birth rates per initiated cycle in patients older than 42 years who underwent IVF treatment with their own eggs were 10.9 % and 9.6 %, respectively, for 43 years and 10.9% and 3.6 %, respectively, for 44 years (Cetinkaya *et al.*, 2013). Patients older than 43 years who use their own eggs generally have poor reproductive outcomes, with a birth rate below 5%, regardless of whether the uterine anatomy is normal or abnormal (Hlinecka *et al.*, 2022). The probability of achieving a live birth in patients undergoing IVF/ ICSI cycles with their own eggs aged 43 years and older is low, even in patients whose ovarian reserve can be considered relatively normal for their age. Such patients might need to undergo multiple IVF cycles to achieve a pregnancy (Fernandez *et al.*, 2021), which did not occur with our patient, who became pregnant on her first IVF attempt.

The main strengths of this report are the age of the patient at the time of oocyte retrieval, her desire to use her own oocytes despite her extremely low AMH, which represents overcoming a barrier to achieving a successful pregnancy, and her overall IVF outcome. It is unusual for a woman at this age, with an AMH of 0.02ng/mL, to be able to retrieve five oocytes, of which four were matured and resulted in three day 5 blastocysts. Moreover, she obtained 33% euploidy, which is also extremely rare for her age group (La Marca *et al.*, 2022). Compared to the previously mentioned case reports, our patient had a lower prognosis in terms of ovarian reserve, but a better outcome was achieved. It is speculative whether this performance is related to the use of antioxidants and testosterone prior to ovarian stimulation. As already mentioned, advanced maternal age can be considered a major concern for aneuploidy and genetic disorders in offspring based on the context of a growing number of women postponing maternity at increasingly older ages. Our patient produced one euploid blastocyst available for transfer, although studies emphasize the increase in the embryo aneuploidy rate in patients older than 35 years. The chance of producing a chromosomally normal blastocyst may be even lower than 5% in women older than 43 years (Ubaldi *et al.*, 2019; Mikwar *et al.*, 2020; Charalambous *et al.*, 2023).

Age-related changes in oocyte quantity and quality, in addition to embryo quality, have a negative impact on pregnancy outcomes, confirming the relevance of this case report. Consequently, patients over 40 years old seeking to become pregnant are recommended to receive IVF treatment even if they have high AMH levels (Hou *et al.*, 2023).

Current ASRM guidelines (2013-present) suggest that women over 50 years old should only undergo embryo transfer after proper medical counseling. Women over 55 years of age should be discouraged from any embryo transfer. In summary, women of advanced maternal age who wish to use their own oocytes should be well counseled (Ethics Committee of American Society for Reproductive Medicine, 2012; Wu *et al.*, 2022).

## CONCLUSION

This is a rare case report of a 48-year-old woman who achieved a live birth in her first IVF attempt with autologous oocytes and one euploid blastocyst. Limited attempts at assisted reproductive technology with older women’s own oocytes may be an option in specific cases. It is essential that the couple be appropriately counseled regarding the chance of achieving a pregnancy using autologous oocytes and the possibility of seeking a donor oocyte program in view of poor outcomes associated with advanced maternal age.
